# CenH3 evolution in diploids and polyploids of three angiosperm genera

**DOI:** 10.1186/s12870-014-0383-3

**Published:** 2014-12-30

**Authors:** Rick E Masonbrink, Joseph P Gallagher, Josef J Jareczek, Simon Renny-Byfield, Corrinne E Grover, Lei Gong, Jonathan F Wendel

**Affiliations:** Department of Ecology, Evolution, and Organismal Biology, Iowa State University, Ames, IA 50011 USA

**Keywords:** Diversifying selection, *CenH3* evolution, Alternative splicing, Centromeres, *Gossypium*, *Brassica*, *Oryza*

## Abstract

**Background:**

Centromeric DNA sequences alone are neither necessary nor sufficient for centromere specification. The centromere specific histone, *CenH3*, evolves rapidly in many species, perhaps as a coevolutionary response to rapidly evolving centromeric DNA. To gain insight into *CenH3* evolution, we characterized patterns of nucleotide and protein diversity among diploids and allopolyploids within three diverse angiosperm genera, *Brassica*, *Oryza*, and *Gossypium* (cotton), with a focus on evidence for diversifying selection in the various domains of the *CenH3* gene. In addition, we compare expression profiles and alternative splicing patterns for *CenH3* in representatives of each genus.

**Results:**

All three genera retain both duplicated *CenH3* copies, while *Brassica* and *Gossypium* exhibit pronounced homoeologous expression level bias. Comparisons among genera reveal shared and unique aspects of *CenH3* evolution, variable levels of diversifying selection in different *CenH3* domains, and that alternative splicing contributes significantly to *CenH3* diversity.

**Conclusions:**

Since the N terminus is subject to diversifying selection but the DNA binding domains do not appear to be, rapidly evolving centromere sequences are unlikely to be the primary driver of *CenH3* sequence diversification. At present, the functional explanation for the diversity generated by both conventional protein evolution in the N terminal domain, as well as alternative splicing, remains unexplained.

**Electronic supplementary material:**

The online version of this article (doi:10.1186/s12870-014-0383-3) contains supplementary material, which is available to authorized users.

## Background

The centromere is a specific region of the eukaryotic chromosome that is the assembly point of the kinetochore, a group of proteins that act as a tether for microtubules during cell division. Although eukaryotic centromeres have highly conserved machinery for chromosome segregation, centromere sequences and binding proteins specific to centromeric chromatin are highly variable, even among closely related taxa [1,2]. Specific retroelements and highly homogenized tandem repeats are common in the DNA of eukaryotic centromeres [3,4], though these too apparently diverge rapidly among closely related species [2,5,6]. Appropriate recruitment of these potentially co-evolving molecular components to the same site is enigmatic, as is their mechanism of spread and homogenization among chromosomes. Explanations for the apparent paradox between conservation of function but variability in sequence typically invoke an interplay between centromere function, centromere sequences, and epigenetic factors, such that DNA sequences *per se* become less functionally constrained [2,6,7]. Accordingly, it is generally accepted that centromeres are specified epigenetically [4].

The evolution of *CenH3* is of particular interest due to its centrality in centromere specification and function [8]. Unlike its highly conserved counterpart (histone H3), *CenH3* has extensive sequence variability, particularly in two regions: (1) the non-canonical NH2 terminal tail, (2) the longer loop 1 region [9,10]. DNA sequence diversity as well as alternative splicing can both play roles in generating diversity, which is thought to compensate for the fast-evolving centromeric DNA to ensure consistent centromeric function, although centromeric sequences are neither necessary nor sufficient for kinetochore assembly [2,11].

Given the twin observations of rapid sequence homogenization of centromeric repeats among chromosomes within species, yet rapid divergence among repeats between species, the question arises as to the fate of centromeric repeats, and *CenH3* evolution, in allopolyploids. Polyploidy plays an integral role in the evolution of many organisms, particularly plants [12-14]. The consequences of polyploidy are often extensive [15-23], and the resulting duplicated genes have myriad possible fates [18,24], including gene loss. Patterns of gene retention and loss following genome duplication have been extensively studied with respect to their broad classifications [25-29]. Because two presumably divergent suites of centromeric sequences become reunited into common nucleus at the time of allopolyploid formation, it is intriguing to investigate the subsequent evolutionary dynamics of the centromeric repeats as well as the now duplicated *CenH3* sequences. In most modern diploids, *CenH3* appears to have returned to single copy status following paleopolyploidy events [30], with a few notable exceptions [31]. In contrast, recent allopolyploids often have multiple *CenH3* gene copies [30-32].

Here we evaluate the fate of *CenH3* in allopolyploids from three divergent genera (*Brassica*, *Oryza*, and *Gossypium*) to address the question of whether the evolution of *CenH3* is similar across a broad range of angiosperm taxa. The genera selected contain a diversity of allopolyploid species having either monophyletic or polyphyletic origins. The *Brassica* genus contains three diverse and widely cultivated diploid species (genomes designated A – C) and three allopolyploid species resulting from independent polyploidization events (BBCC, AACC, AABB) [33], while *Oryza* contains multiple diploid genome groups (designated A – G) and allopolyploids of diverse genomic origin (BBCC, CCDD, and HHJJ) [34]. *Gossypium* includes 45 diploid species divided into genome groups designated A – G and K [19], as well as a single, monophyletic [35] polyploid clade (AD genome) containing 6 species. Previous work on *CenH3* in *Brassica* and *Oryza* allopolyploids has focused on selection in specific regions of the gene and the relative expression of the retained homoeologs [30,32]. Less is known about *CenH3* and centromeric evolution in allopolyploid *Gossypium*. A prior study reported a centromeric gypsy-like retroelement (CRG) present in all centromeres of both the allotetraploid *G. hirsutum* (AD-genome) and the model progenitor D-genome diploid *(G. raimondii*), but absent from A-genome species [36]. Neither the sequence nor the expression of duplicated *CenH3* have been evaluated.

We characterize *CenH3* sequence evolution on in three phylogenetically disparate angiosperm genera containing diploids and allopolyploids, and assess patterns of molecular evolution. We address whether allopolyploids retain duplicated copies following allopolyploid formation; the dynamics of sequence evolution of the duplicated, newly co-resident sequences; and the relative expression levels of homoeologous copies. In addition to reporting on *CenH3* sequence evolution within and between genera, we describe novel patterns of alternative splicing in *CenH3*.

## Results

We cloned and sequenced the *CenH3* genes from 7 diploid and 5 allotetraploid *Gossypium* species. The length of genomic sequences varied from 2565 to 2673 bp in the diploids and 2654 to 2673 bp in the polyploids (Additional file 1), although protein-coding length was consistent for all species, 492 bp. While length variation was not detected among cDNAs, we cannot account for length variation that occurs outside of our external primers in the first and last exons. The structure of *CenH3*, 7 exons and 6 introns, was conserved among all polyploid species, (diploid cDNA sequences were inferred from genomic sequences). As expected, *CenH3* was largely conserved across the genus, with the majority of polymorphisms occurring in introns. *Gossypium exiguum* exhibited the greatest difference observed among the diploids (93 bp deletion in intron 6); the remainder of the polymorphisms were small (<10 bp), five of which were phylogenetically informative (Additional file 2).

**Figure 1:**
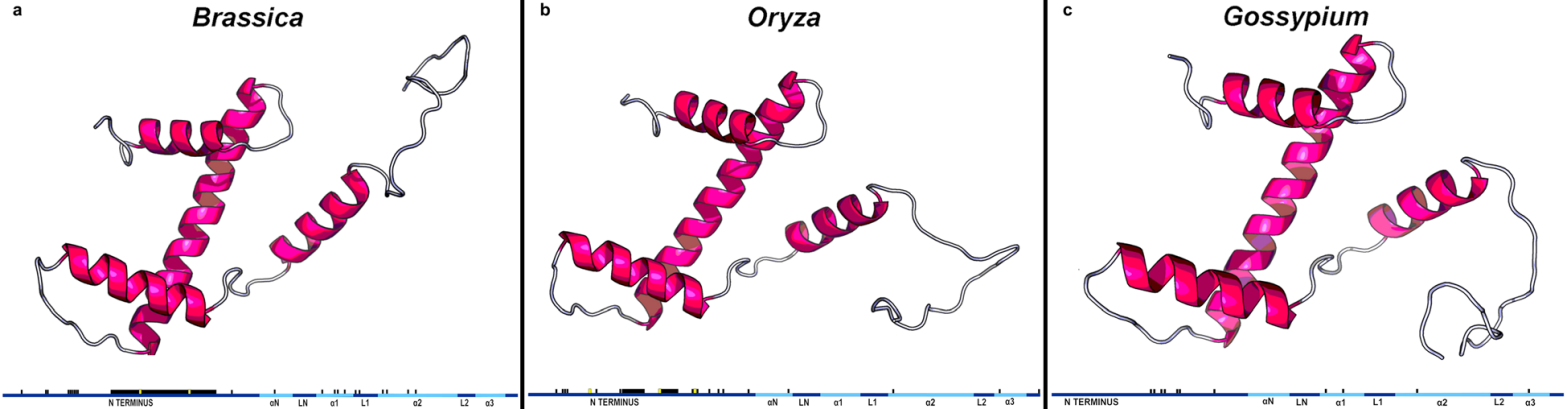
**3D structures of CenH3 proteins from a single species in each genus. a**. *Brassica napus (AC8)*. **b**. *Oryza australiensis isolate (EE)*. **c**. *Gossypium raimondii (D5).* The secondary protein structures are depicted below each 3D model containing nonsynonymous SNPs within each genus mapped to the secondary structure of the CenH3 protein. The different shades of blue signify the borders of each protein domain; α signifies an alpha helix and L signifies a loop. Black rectangles signify nonsynonymous SNPs, while yellow rectangles signify nonsynonymous SNPs under positive selection (Figure 3). The N terminus was the primary site of length variation among the three genera and the long black rectangle in the N terminus of the *Brassica CenH3* signifies gaps in the alignment among *Brassica* species.

Since CenH3 is thought to co-evolve with rapidly changing centromeric DNA, we mapped nonsynonymous substitutions to the secondary protein structure for three genera (*Brassica*, *Oryza*, and *Gossypium*) (Figure 1) in order to localize evolution along the protein. The N terminus was the only domain with high levels of nonsynonymous substitutions that was consistent between all three genera. *Brassica* had the highest numbers of nonsynonomous substitutions, which was followed by *Oryza* and then *Gossypium*. This observation is in agreement with previous analyses which show that rates of molecular evolution are faster in herbaceous plants (*Oryza* and *Brassica*) than in trees and shrubs (*Gossypium*) [37].

**Figure 2:**
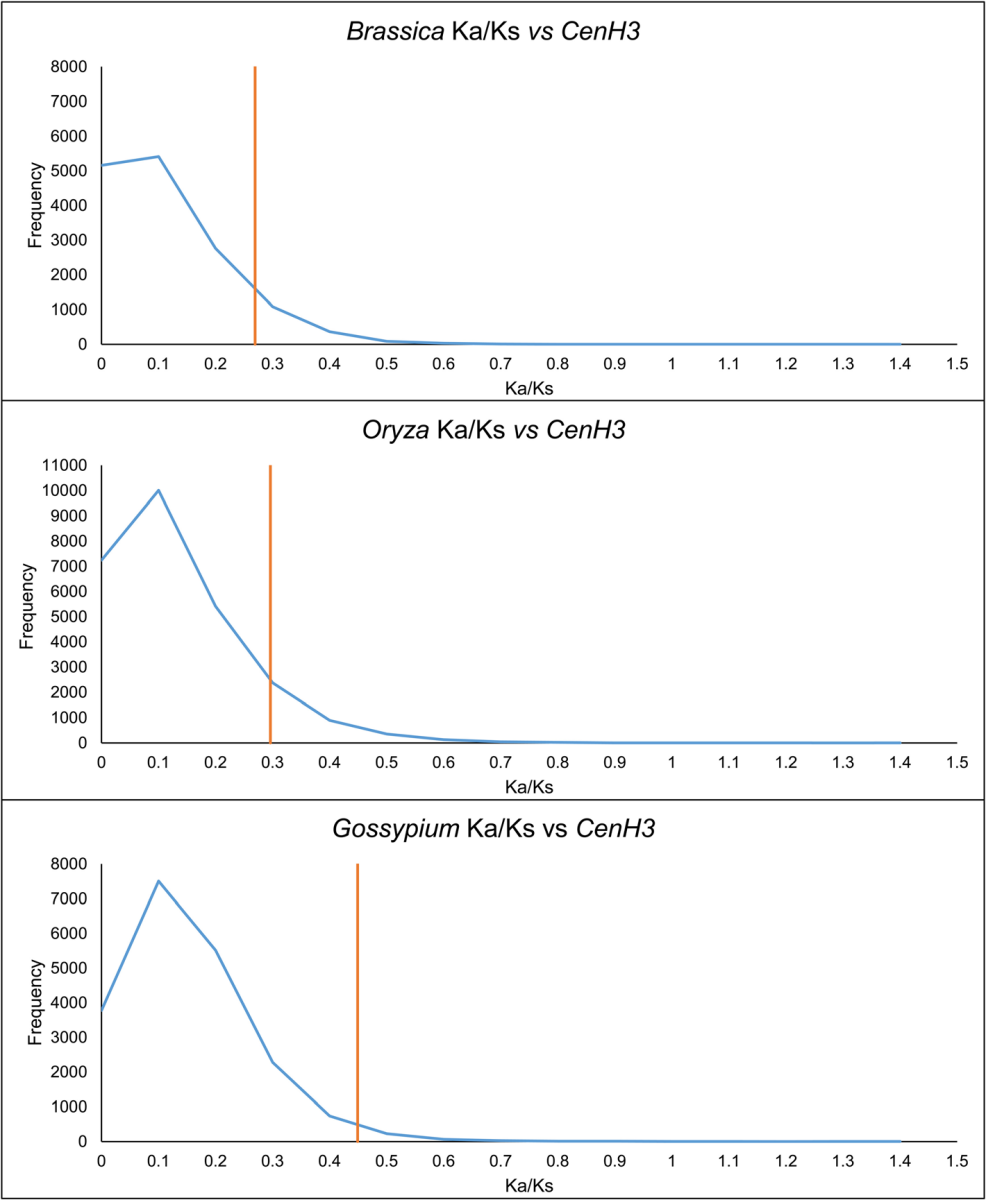
**The Ka/Ks ratio for the CDS from a representative species of** ***Brassica, Oryza,*** **and** ***Gossypium.*** Each representative species was used to compare the rate of *CenH3* evolution to other genes in each genus. The y-axis is the number of genes that correspond to the Ka/Ks bin values on the x-axis. The blue line is the whole genome Ka/Ks, and the orange line is *CenH3* from each taxon.

To determine the extent of *CenH3* evolution, we compared Ka/Ks (nonsynonymous/synonymous substitutions) ratios for all CDSs of a species from each genus with respect to an outgroup. *CenH3* is indeed a relatively fast evolving gene, with the *Brassica*, *Oryza*, and *Gossypium* genes falling in the 81st, 85th, and 97th percentile of all genes in these genomes, respectively (Figure 2).

**Figure 3:**
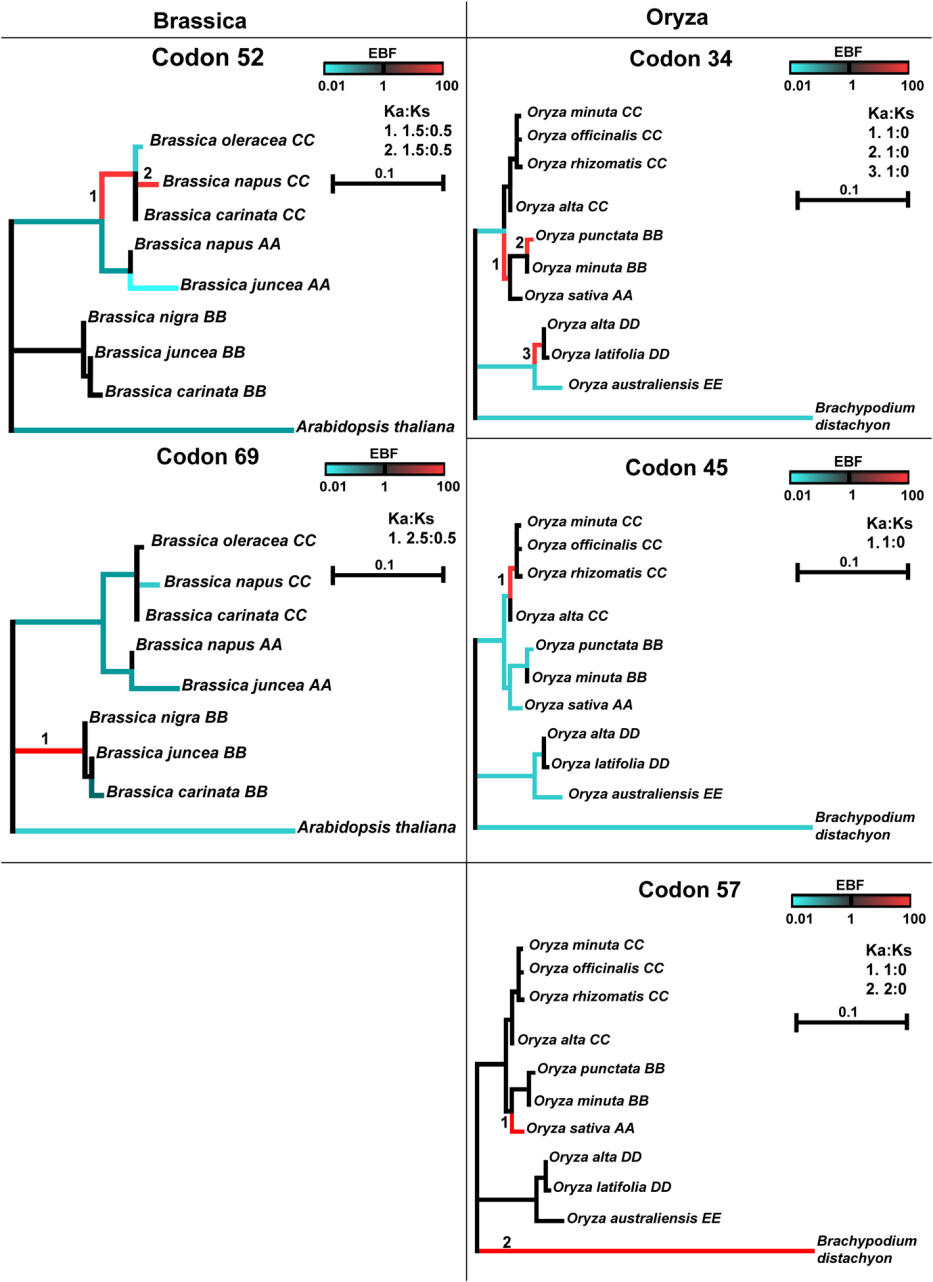
**Episodic diversifying selection in** ***Brassica*** **and** ***Oryza*** **, as inferred using MEME.** Each phylogeny represents a single codon exhibiting diversifying selection in the specified genus. The scale bar represents distance and EBF is the empirical Bayes factor, which signifies diversifying selection with warm colors and stabilizing selection with cool colors. Particular branches are labeled with numbers, which correspond to the Ka:Ks of a single codon on the right of each phylogeny.

To quantify *CenH3* evolution in each genus we estimated the mean Ka/Ks of *CenH3* using DNAsp (Table 1) [38]. The overall mean Ka/Ks ratios within each genus and between each genus and an outgroup all indicate that diversifying selection is absent from the *CenH3* gene as a whole. To determine whether this finding is consistent at a finer scale, we used the mixed effects model of evolution software (MEME) [39] to evaluate selection at the codon level in each genus (Table 2, Figure 3). In *Brassica* and *Oryza*, the only codons with evidence suggestive of diversifying selection were limited to the N terminus, while no evidence of diversifying selection was found for *Gossypium*. Inferred codons subject to selection in *Oryza* and *Brassica* were phylogenetically episodic and specific to only a few species for each codon (Figure 3).

**Table 1:** **Jukes Cantor corrected estimates of Ka/Ks within each genus, and between each genus and outgroup**

	**Within**			**Between**		
	***Brassica***	***Oryza***	***Gossypium***	***Brassica***	***Oryza***	***Gossypium***
Ka	0.045	0.029	0.008	0.143	0.218	0.025
Ks	0.126	0.132	0.020	0.550	0.600	0.039
Ka/Ks	0.354	0.217	0.401	0.260	0.390	0.643

**Table 2:** **Significant p-values associated with codons in** ***Brassica*** **and** ***Oryza*** **, as inferred from MEME analysis**

**Genus/codon**	**P-value**
*Brassica*/52	0.0379
*Brassica*/69	0.009
*Oryza*/34	0.008
*Oryza*/45	0.030
*Oryza*/57	0.045

### *CenH3* evolution in allopolyploids

Given the single copy status observed in most diploid angiosperms sequenced to date, despite an evolutionary history, which encompasses multiple episodes of polyploidy, we wished to address whether or not *CenH3* has been retained in duplicate following recent allopolyploidy events. In *Gossypium*, we found that each diploid representative had a single *CenH3* gene and that all allopolyploid species had two, indicating retention of both parental copies, as reported for *Brassica* and *Oryza* [30,32,40].

To address whether or not duplicated *CenH3* sequences evolve independently of one another following allopolyploid formation, or if instead they are subjected to some form of sequence interaction or homogenization, we manually analyzed *CenH3* sequences from all three genera. This lack of independence has been demonstrated for other homoeologous single copy genes in allopolyploids, most notably in *Gossypium* where the phenomenon was first described [41,42]. *CenH3* gene conversion was absent in all three genera, which is consistent with previous reports in *Brassica* and *Oryza* [30,32].

Genomic DNA sequences for *CenH3* from *Gossypium* were used to construct a maximum likelihood tree, which concurs with the currently accepted phylogeny for the genus (Additional file 3). We resolved monophyletic clades for both the A_T_ and D_T_ homoeologs and their respective model diploid progenitors. Gene conversion was not detected in the homoeologs, further confirming that independent evolution of *CenH3* homeologs occurred in this 1–2 MYD polyploid clade.

### *CenH3* gene expression

As mentioned above, there are various possible fates for genes duplicated via polyploidy. While both parental copies of *CenH3* were retained in all studied allopolyploid species of *Oryza*, *Brassica*, and *Gossypium*, the transcriptional usage of each parental copy can vary from equivalent to complete silencing of one parental copy. To assess expression of *CenH3* in *Gossypium,* we used three independent methods that allow us to assess the relative expression of homoeologs. The same tissue source was used for clone-counting and chromatogram measurements, while RNA-seq data from different sources was used to investigate other aspects of CenH3 expression. The RNA-seq data sources included (A2 vs D5) to determine the relative expression of *CenH3* in the model diploid progenitors to the cotton polyploids, a synthetic hybrid between these two diploids (A2XD5F1), a synthetic polyploid (2_A2D1), and a domesticated and wild accession of AD1 (maxxa and yuc respectively).

**Figure 4:**
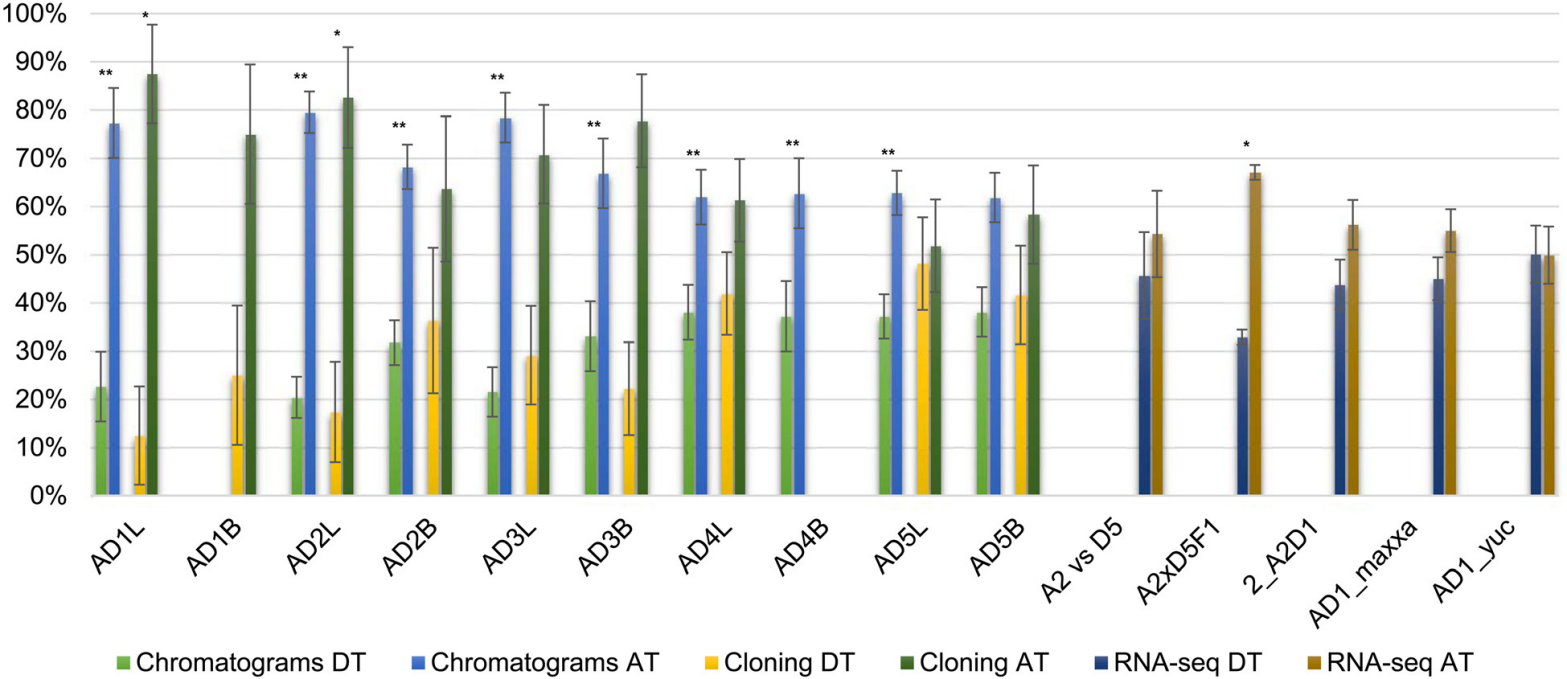
**Homoeolog expression of** ***CenH3*** **in** ***Gossypium*** **species.** AD1-AD5 denote *G. hirsutum, G. barbadense, G. tomentosum, G. mustelinum,* and *G. darwinii*, respectively. The L and B suffixes denote leaf and bud tissue, respectively. A2 vs D5 is a comparison of the total level of expression in the model diploid progenitor species, *G. arboreum* (A) and *G. raimondii* (D). A2xD5F1 is an F1 hybrid between *G. arboreum* and *G. raimondii*. 2_A2D1 is a colchicine-doubled F1 hybrid between *G. arboreum* (A) and *G. thurberi* (D). AD1_maxxa and AD1_yuc are domesticated and wild accessions of *G. hirsutum*, respectively. Only partial data were generated for AD1B and AD4B. Standard deviations are represented by the error bars. A single asterisk represents samples that were statistically significant below 0.05, while two asterisks represent significant below 0.01.

The three methods to analyze expression resulted in differing degrees of homoeolog bias (where A_T_ and D_T_ are used to denote the two homoeologs), which was moderate in the RNA-seq data, and more extreme in the other methods (Figure 4). A_T_ homoeolog, expression was favored in every species, tissue, and test (Figure 4). With RNA-seq data we compared the total expression levels of the model progenitor diploids (A2 vs D5), a synthetic polyploid (2(A2D1)), and wild and domesticated accessions of AD1 (yucatanense and Maxxa, respectively), all of which lacked a significant difference in expression. The only sample with a significant expression bias was the F1(A2D5), biased at 87.5% (P ≤ 0.05). The difference in homoeolog expression was not significantly different (T test) between leaf and bud tissue, except that A_T_ homoeolog expression was significantly higher in leaves for *G. barbadense* (P = 1.1024 × 10^−10^).

Due to the sequence similarity between homoeologs, specific primers could not be designed for quantitative PCR, and thus we measured homoeolog expression bias with three separate methods, accompanied by different caveats. Due to the low number of SNPs between the homoeologs, a large number of RNA-seq reads could not be allocated to a particular homoeolog. We also cloned *CenH3* cDNAs, which were counted to calculate relative expression of homoeologs, albeit with a smaller sample size (Additional file 4).

### Alternative splicing

**Figure 5:**
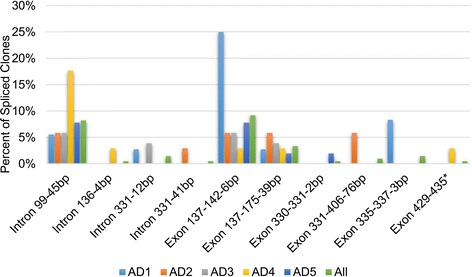
**Frequency of splicing variants in all** ***Gossypium*** **allopolyploids.** Splicing variants found from cloning PCR amplified cDNAs and displayed as a percent of the total number of clones obtained from each species. For the “All” category the splicing variants are displayed as a percent of the total number of clones obtained for all species combined. Exon stands for exon deletion and Intron denotes an intron retention. The asterisk on EXON 429–435 stands for unknown, since the PCR product ended at nucleotide position 435. AD1-AD5 denote different polyploid species in numerical order: *G. hirsutum, G. barbadense, G. tomentosum, G. mustelinum,* and *G. darwinii*. CenH3 protein secondary structure is aligned to the nucleotide positions of the cDNA below.

Alternative splicing of transcripts is one mechanism by which novel proteins are created, which conceivably may provide the diversity for interaction between rapidly evolving partners like CenH3 and centromeric DNA. To address how alternative splicing affects *CenH3*, we assessed the level of alternative splicing for each homoeolog of *CenH3* in allopolyploid *Gossypium* by cloning and sequencing the amplified *CenH3* cDNAs from leaf and leaf bud tissues. The sum frequency of alternatively spliced transcripts for both tissues was 26.7% (55/206 transcripts) and consisted of 33 exon deletions and 22 intron retentions (relative to the major variant) for all allopolyploids. Three splicing variants were found in all species evaluated (Figure 5): (1) 45 bp intron retention at position 99 (8.3% of transcripts); (2) a 6 bp exon deletion from position 137–142 (9.2% of transcripts); and (3) a 39 bp exon deletion in A_T_ homeologs from 137–175 (3.4% of all transcripts or 4.9% of A_T_ transcripts; Figure 5). One splicing variant, a 12 bp intron retention, was shared only between *G. hirsutum* (AD1) and *G. tomentosum* (AD3) (Figure 5). The most common splicing variants resulted in either a slight deletion or extension of the N terminus, thus adding diversity to an already rapidly evolving domain of *CenH3*, while the least common splicing variants resulted in nonfunctional protein predictions.

*G. hirsutum* (AD1) had the highest frequency of spliced transcripts at 44.4% (Additional file 5). Each species had a different proportion of each splicing variant; for example, the exon 137–142 deletion was present in 25*%* of *G. hirsutum* (AD1) clones, while it was present at 2.9-7.8% of other species. 17.6% of *G. mustelinum* (AD4) clones included the 45 bp intron insertion at position 99, which was only present 5.6-7.8% in the other species (Figure 5).

## Discussion

Despite its crucial role in centromere specification, many aspects of *CenH3* evolution are poorly understood. Recent years have brought advances in our understanding of centromere epigenetics and evolution. For example, tandem repeats in many species have an evolutionary relationship with the CenH3 protein to reduce nucleosomal bending energy [43], multiple proteins interact with centromeric DNA to induce positive supercoiling of centromeric DNA [44], and CenPA (CenH3 in plants) provides the foundation for binding other kinetochore proteins [45].

### *CenH3* sequence evolution in angiosperms

The present study extends our knowledge of the pace and process of *CenH3* evolution by evaluating genomic and expression changes in three diverse angiosperm genera. Previous research in *Brassica and Oryza* showed that the *CenH3* N terminus and CATD (loop 1 and α2 helix domains) sequences were under diversifying selection in lineage specific manners [30,32]*.* To assess the generality of these findings in angiosperms, we reanalyzed the *Oryza* and *Brassica* sequences at a finer scale to identify specific regions of *CenH3* that have actively diversified, and performed the first *CenH3* sequence analysis for *Gossypium*. Across all three genera, diversification generally occurs in the N terminus, a result consistent with previous reports of rapid evolution in this domain, but contrary to reports of diversification in the CenPA Targeting Domain [10,46-50].

### Retention and expression of *CenH3* alleles in allopolyploids

In all three genera, both homoeologous copies are retained following genome doubling, demonstrating that restoration to single copy status, as widely observed among modern “diploid” plants, need not occur quickly following WGD events. Interestingly, expression of homoeologous *CenH3* copies in *Gossypium* exhibited directional bias in all samples, although differences were not always statistically significant (Figure 4); this result contrasts with reports from *Brassica* and *Oryza* allopolyploids [30,32]. In *Brassica* allotetraploids, a variety of *CenH3* expression patterns were found for homoeologs, from a 2:1 ratio in an accession of *B. juncea* to complete B-genome *CenH3* suppression in an accession of *B. carinata* [32]. In allotetraploid *O. minuta* and *O. alta*, *CenH3* expression is unbiased [30]. The variation in expression profiles among allopolyploids in these three genera is notable and without an obvious explanation, although it seems likely that homoeolog expression levels reflect the unique genomic and evolutionary idiosyncrasies that characterize hybridization and genome doubling in each genus.

### Alternative splicing of *CenH3*

In addition to non-synonymous evolution, protein diversity may be generated by alternative splicing. Considering the three genera collectively, it appears that alternative splicing frequently modifies the N terminus of *CenH3* in *Brassica* and *Gossypium*, and this is the only domain modified in *Oryza* [30,32]. Interestingly, the N terminus often cannot be aligned among closely related genera, yet it is necessary for centromeric deposition of *CenH3* during meiosis in *A. thaliana* [51,52]. The N terminus also interacts with kinetochore proteins in *S. cerivisiae* [53]. Perhaps alternative splicing represents another means to generate the diversity of sequences necessary for CenH3 to target centromeres in meiosis, or a rapid defense that introduces new CenH3 proteins in response to increased centromere size. An alternative is that alternative splicing can also lead to a differing abundance of alternate transcripts between cells and tissues, which has implications in centromeric DNA if *CenH3* and centromeric DNA are indeed coevolving.

## Conclusions

By comparing the *CenH3* sequences from three disparate angiosperm genera, we have gained insight into the rates and regions of evolution in this important protein. The most commonly mutated domain is the N terminus, which is also subject to alternative splicing, and contributes significantly to diversity in the N terminus of *Brassica* and *Gossypium* CenH3s*.* Alternative splicing is largely absent from the histone fold domain, even though loop 1 and α2 helix domains bind centromeric DNA, which therefore likely are subject to length constraints.

While CenH3 is considered a rapidly evolving protein, the N terminus is the only domain that is unalignable among closely related genera and is the most diverse domain. Roles have been attributed to the N terminus of *CenH3*, such as ubiquitin-mediated proteolysis [53] and it is indispensable for meiotic localization of CenH3, yet the domain is expendable in mitosis [51,52]. In humans the N terminus is a hotspot for posttranslational modifications that interact with other centromeric proteins [54], and perhaps this is the case in plants as well. At present, the functional explanation for the diversity generated by both conventional protein evolution in the N terminal domain, as well as alternative splicing, remain unexplained.

The basis for the interest in CenH3 is its ability to bind to rapidly evolving centromeric repeats and yet still interact with conserved elements in the kinetochore. The dynamics of this relationship at the polyploid scale are increasingly complex due to the duplication of *CenH3* and exposure to a new regime centromere repeats. The mono and polyphyletic origins of polyploids seem to influence the direction and level of expression bias between *CenH3* homoeologs (Figure 4) and is most pronounced after hybridization, as is seen in the F1(A2D5) of *Gossypium*, but not following polyploidy 2(A2D1). Another compelling issue is obtaining diversity in these transcripts to respond to repeat evolution in the centromere, which is sparse in our tests for selection. Alternative splicing may be an another means to obtain this diversity, although neither selection nor alternative splicing modified the DNA binding domain of *CenH3*. Since the N terminus is the only domain modified by alternative splicing and subject to diversifying selection, but the DNA binding domains are not, rapidly evolving centromere sequences are unlikely to be the primary driver of *CenH3* sequence diversification.

## Methods

### Plant materials

Leaves (2–4 cm) and leaf buds were collected from eight diploid cottons: *G. arboreum* cv. 101 [A2] (ISC427583), *G. anomalum* [B1] (ISC447893)*, G. robinsonii* [C2] ISC451818)*, G. raimondii* [D5] (ISC429440)*, G. stocksii* [E1] (ISC447876)*, G. longicalyx* [F1] (ISC418550)*, G. bickii* [G1] (ISC414834)*, G. exiguum* [K] (ISC416400), and from five allopolyploid cottons (*G. hirsutum* cv. TM1 [AD1] (ISC451819), *G. barbadense* cv. Pima S6 [AD2] (ISC451820), *G. tomentosum* 95 [AD3] (ISC451821), *G. mustelinum* local lab accession [AD4] (ISC429442), and *G. darwinii* PW45 [AD5] (ISC429431). For purposes of phylogenetic reconstruction, we included the outgroup species *Gossypioides kirkii* (ISC 418555) [55]. All lines were grown in the Pohl Conservatory at Iowa State University and were used for both DNA and RNA extractions. DNA was extracted using the Qiagen DNeasy Plant Kit following the manufacturers recommended protocol. RNA was extracted using the Sigma Spectrum RNA Extraction Kit following the manufacturers recommended protocol with the following modifications: protocol A was followed at step 4, one wash each was performed for wash solutions I and II, and on-column DNA digestion was performed with the Sigma On-Column DNase I Digest Set.

### *CenH3* sequencing

*CenH3* gene sequence data for *G. raimondii* was obtained from Phytozome [56,57], from which primers were designed (Additional file 6) for PCR amplification and sequencing from other species. PCR amplifications were performed using the manufacturers recommended reaction mixtures/cycling conditions and a melting temperature of 57°C. PCR amplicons from all diploid accessions (except *G. exiguum*) were cleaned via the Qiaquick PCR column cleanup (Qiagen) and sequenced with the amplification primers and a set of internal sequencing primers (Additional file 6).

PCR products from *G. exiguum* and all polyploid species were visualized on an Invitrogen E-gel to isolate bands, and cloned with the P-GEM-T Easy Vector ligation kit (Promega) and Top10 Competent Cells (Invitrogen) according to the recommended protocol. Clones were sequenced at the Iowa State DNA Sequencing Facility using both M13 primers and internal primers (Additional file 6).

*CenH3* cDNA sequences for *Oryza* and *Brassica* were downloaded from GenBank [58] and Phytozome [57] (Additional file 7).

### Evaluation of selection

Jukes Cantor corrected estimates of Ka/Ks were measured using DNAsp. The MEME software package [39] accessed via the (http://www.datamonkey.org) server [59], was used to test for selection at the codon level. The automatic selection tool was used to determine the correct substitution models for *Brassica* (F81), *Gossypium* (F81), and *Oryza* (HKY85). The significance level cutoff was set at P = <0.05. We used *Arabidopsis thaliana* (24 million years divergence (MYD); Lysak et al. [60]), *Gossypioides kirki* (13.6 MYD; Cronn et al. [61]), and *Brachypodium distachyon* (46 MYD; Sanderson [62]) as the outgroups for *Brassica* (7.9 MYD; Jacquemin et al. [63]), *Gossypium* (5–10 MYD; Senchina et al. [64]), and *Oryza* (15 MYD; Sanderson [62]), respectively.

We used the SynMap tool of CoGe (http://genomeevolution.org/CoGe) [65,66] to identify blocks of syntenic orthologs to evaluate whole genome Ka/Ks for a representative species from each genus (*Brassica rapa, Oryza sativa, Gossypium raimondi*). The following parameters were used: BlastN, relative gene order, −D 50, −A 10, quota align merge –Dm 80, quota align with a ratio of coverage depth at 3:1 for (*B. rapa*: *A. thaliana*), 1:1 for (*O. sativa*: *B. distachyon*), and 6:1 for (*G. raimondii*: *Theobroma cacao*), overlap distance 40. Each species was compared to their previously described outgroups, except *T. cacao* (60 MYD) [67] was the outgroup for *G. raimondii,* since *Gossypioides kirki* lacks a sequenced genome.

### Phylogenetic analysis

Genomic DNA sequences were aligned using CLUSTALW [68] in BioEdit [69] and converted to NEXUS format using readal (http://trimal.cgenomics.org). The best fitting model of DNA sequence evolution was determined using the AIC and BIC, as calculated by jModelTest [70,71]. Since both the GTR + Γ model and the HKY + Γ model were favored by AIC and BIC respectively, MEGA6 was used to build bootstrapped maximum likelihood trees with 100 replicates under both models [72,73]. The log likelihood of the GTR + Γ tree was slightly higher and is reported here; however, both trees exhibited a similar topology.

### Protein structure prediction

Secondary and tertiary protein structures were modeled using RaptorX [74-77]. This software compares alignments of the sample protein to other proteins with known structural information to determine a probable structure using statistics. A representative *CenH3* sequence from each genus was modeled (*G.raimondii* (D5), *Oryza australiensis* (EE), and *Brassica napus* (AC8)) (Figure 1).

### cDNA generation and sequencing of *CenH3* transcripts

Reverse transcription was performed using the Invitrogen SuperScript III First-Strand Synthesis System Kit using oligo dT primers, and *CenH3* was amplified from the cDNA pool using primers that were designed from the 5’ and 3’ outermost exons (Additional file 6). PCR products were processed using the Qiaquick PCR Cleanup columns and sequenced with the amplification primers (Additional file 6).

Chromatogram-based expression estimates were calculated as described previously [78]. At least three replicates were used for each tissue to permit standard error calculations and paired, two-tailed T tests were used to test for significance. Expression levels for the polyploid accessions were secondarily estimated with RNA-seq data and by cloning cDNA amplicons (as described above). The clones were randomly selected from each sample, sequenced, and then grouped by their subgenomic origin “A_T_” and “D_T_”. Since the samples should follow a binomial distribution, the null hypothesis for the rate of cloning each homeologous copy of CenH3 should be 0.5. To control for the FWER (Family-wise Error Rate) at α = 0.05, the Bonferroni correction was determine the significance.

### Expression estimation via RNA-seq

To assess *CenH3* gene expression, we analyzed previously generated leaf transcriptome data (SRA BioProject PRJNA171342) [79] for both model diploid parents, an F1 hybrid of *G. arboreum* (A2 genome) and *G. raimondii* (D5 genome), a colchicine doubled F1 hybrid of *G. arboreum* and *G. thurberi* (D1 genome), and two accessions of the allopolyploid (AD genome) *G. hirsutum* (*G. hirsutum* var yucatanense, a wild accession; *G. hirsutum* cv Maxxa, a domesticated accession). Raw reads were trimmed with sickle (https://github.com/najoshi/sickle), and mapped to the generated *CenH3* sequences using GSNAP (batch 4, novel splicing on) [80] in conjunction with a *CenH3*-specific SNP index to efficiently map sequences from different species and subgenomes. The SNP indices were manually curated from Sanger sequencing of the cDNA’s and gene sequences. For sequences from the hybrid and polyploid, PolyCat [81] was used to partition A- and D-genome derived reads. The significance for homeolog bias was calculated using a paired, Student’s T-test with log2 transformation to ensure the normality in expression values.

### Availability of supporting data

CenH3 gene sequence data have been submitted to GenBank. Accession numbers can be found in Additional file 1.

## Additional files

Additional file 1:
**Genomic sequence lengths of CenH3 in** ***Gossypium*** **and Gossypioides.** *Gossypium CenH3* accession numbers and gene lengths. Additional file 2:
**Phylogenetically informative alternative splicing variants in** ***Gossypium*** **allopolyploids.** Splicing variants of *Gossypium* grouped informatively. Additional file 3:
***Gossypium*** **CenH3 Phylogeny.** A CenH3 phylogeny of *Gossypium* diploids and polyploids. Phylogeny of *Gossypium* using genomic *CenH3* sequences. The *Gossypium* phylogeny is in agreement with previously published phylogenies [19], excluding the polytomy seen in the AT sub-tree, which was the result of too few informative SNPs in the CenH3 gene. A is *G. arboreum*, B is *G. anomalum*, C is *G. robinsonii, D is G. raimondii*, E is *G. stocksii*, F is *G. longicalyx*, G is *G. bickii*, K is *G. exiguum* [K], AD1 is *G. hirsutum*, AD2 is *G. barbadense*, AD3 is *G. tomentosum*, AD4 is *G. mustelinum, AD5 is G. darwinii*, Gk is *Gossypioides kirkii*. A scale bar is provided at the bottom; numbers at each node are bootstrap support values. Additional file 4:
**The number of clones for each sub-genome in allopolyploid** ***Gossypium*** **.** Counts of CenH3 clones allocated to each subgenome in *Gossypium* allopolyploids. Additional file 5:
**Percentage of clones with splicing variations in each polyploid genome.** The percentage of the total number of clones that contained splicing variants in each polyploid genome. Percent of splicing variants found in clones from each species. Total number of clones from each species was the sum total of the clones from bud and leaf tissue, if both were available. AD1-AD5 denote *G. hirsutum, G. barbadense, G. tomentosum, G. mustelinum,* and *G. darwinii*, respectively. Additional file 6:
**Primer sequences for genomic and cDNA amplification.** Primer sequences. Additional file 7:
**Accessions downloaded from GenBank and Phytozome.** Species names and accession numbers of *Brassica* and *Oryza* CenH3 sequences.
