# Supplementary material for: CenH3 evolution in diploids and polyploids of three angiosperm genera
Source: BMC Plant Biol. 2014 Dec 30;14:383. doi: 10.1186/s12870-014-0383-3 (PMC4308911; doi:10.1186/s12870-014-0383-3)
Supplement: Additional file 1: — Genomic sequence lengths of CenH3 in Gossypium and Gossypioides. Gossypium CenH3 accession numbers and gene lengths. [file 12870_2014_383_MOESM1_ESM.docx]

**Additional file 1** Genomic sequence lengths of *CenH3* in *Gossypium* and *Gossypioides*

| *Species* | Accession | Genome | Genomic DNA (bp) |
| --- | --- | --- | --- |
| *G. arboreum* | KP177465 | A | 2673 |
| *G. anomalum* | KP177457 | B | 2664 |
| *G. robinsonii* | KP177458 | C | 2656 |
| *G. raimondii* | KP177464 | D | 2657 |
| *G. stocksii* | KP177459 | E | 2672 |
| *G. longicalyx* | KP177460 | F | 2669 |
| *G. bickii* | KP177461 | G | 2664 |
| *G. exiguum* | KP177462 | K | 2565 |
| *G. hirsutum* – A_T_ | KP177466 | AD1 | 2654 |
| *G. barbadense* – A_T_ | KP177468 | AD2 | 2670 |
| *G. tomentosum* – A_T_ | KP177469 | AD3 | 2664 |
| *G. mustelinum* – A_T_ | KP177470 | AD4 | 2666 |
| *G. darwinii –* A_T_ | KP177467 | AD5 | 2669 |
| *G. hirsutum* – D_T_ | KP177475 | AD1 | 2660 |
| *G. barbadense* – D_T_ | KP177472 | AD2 | 2658 |
| *G. tomentosum* – D_T_ | KP177471 | AD3 | 2660 |
| *G. mustelinum* – D_T_ | KP177474 | AD4 | 2668 |
| *G. darwinii* - D_T_ | KP177473 | AD5 | 2660 |
| *Gossypioides kirkii* | KP177463 |  | 2665 |
