# Supplementary material for: CenH3 evolution in diploids and polyploids of three angiosperm genera
Source: BMC Plant Biol. 2014 Dec 30;14:383. doi: 10.1186/s12870-014-0383-3 (PMC4308911; doi:10.1186/s12870-014-0383-3)
Supplement: Additional file 2: — Phylogenetically informative alternative splicing variants in Gossypium allopolyploids. Splicing variants of Gossypium grouped informatively. [file 12870_2014_383_MOESM2_ESM.docx]

**Additional file 2** Phylogenetically informative alternative splicing variants in *Gossypium* allopolyploids.

| Subject | Event |
| --- | --- |
| G. robinsonii, G. bickii, and G. exiguum | 9bp deletion in intron 2 |
| G. barbadense and G. darwinii | 9bp deletion in intron 4 |
| Dt homeologs G. tomentosum (2)and G. hirsutum (2), G. barbadense (3), G. mustelinum (3), G. darwinii (3) | 7bp microsatellite copy number variant in intron 6 |
| All Dt homeologs (2) | 3bp deletion in intron 6 |
| Event conserved in G. raimondii and all Dt homoeologs | 9bp in intron 4 |
