# Supplementary material for: CenH3 evolution in diploids and polyploids of three angiosperm genera
Source: BMC Plant Biol. 2014 Dec 30;14:383. doi: 10.1186/s12870-014-0383-3 (PMC4308911; doi:10.1186/s12870-014-0383-3)
Supplement: Additional file 4: — The number of clones for each sub-genome in allopolyploid Gossypium . Counts of CenH3 clones allocated to each subgenome in Gossypium allopolyploids. [file 12870_2014_383_MOESM4_ESM.docx]

**Additional file 4**  The number of clones for each sub-genome in allopolyploid *Gossypium*.

|  | DT Clones | AT Clones | Total |
| --- | --- | --- | --- |
| AD1L | 3 | 21 | 24 |
| AD1B | 3 | 9 | 12 |
| AD2L | 4 | 19 | 23 |
| AD2B | 4 | 7 | 11 |
| AD3L | 7 | 17 | 24 |
| AD3B | 6 | 21 | 27 |
| AD4L | 13 | 21 | 34 |
| AD5L | 13 | 14 | 27 |
| AD5B | 10 | 14 | 24 |

AD1-AD5 denote *G. hirsutum, G. barbadense, G. tomentosum, G. mustelinum,* and *G. darwinii*, respectively. The L and B suffixes correspond to leaf and bud tissue respectively. Diagnostic SNPs were used to assign each clone to the AT or DT homoeolog. All clones are derived from three independent PCR reactions and three independent transformations, except AD1B (1). Only partial data were obtained for *G. mustelinum* leaf bud tissue, and clones were obtained from a single PCR reaction for *G. hirsutum*.
