# Supplementary figures and images for: CenH3 evolution in diploids and polyploids of three angiosperm genera
Source: BMC Plant Biol. 2014 Dec 30;14:383. doi: 10.1186/s12870-014-0383-3 (PMC4308911; doi:10.1186/s12870-014-0383-3)

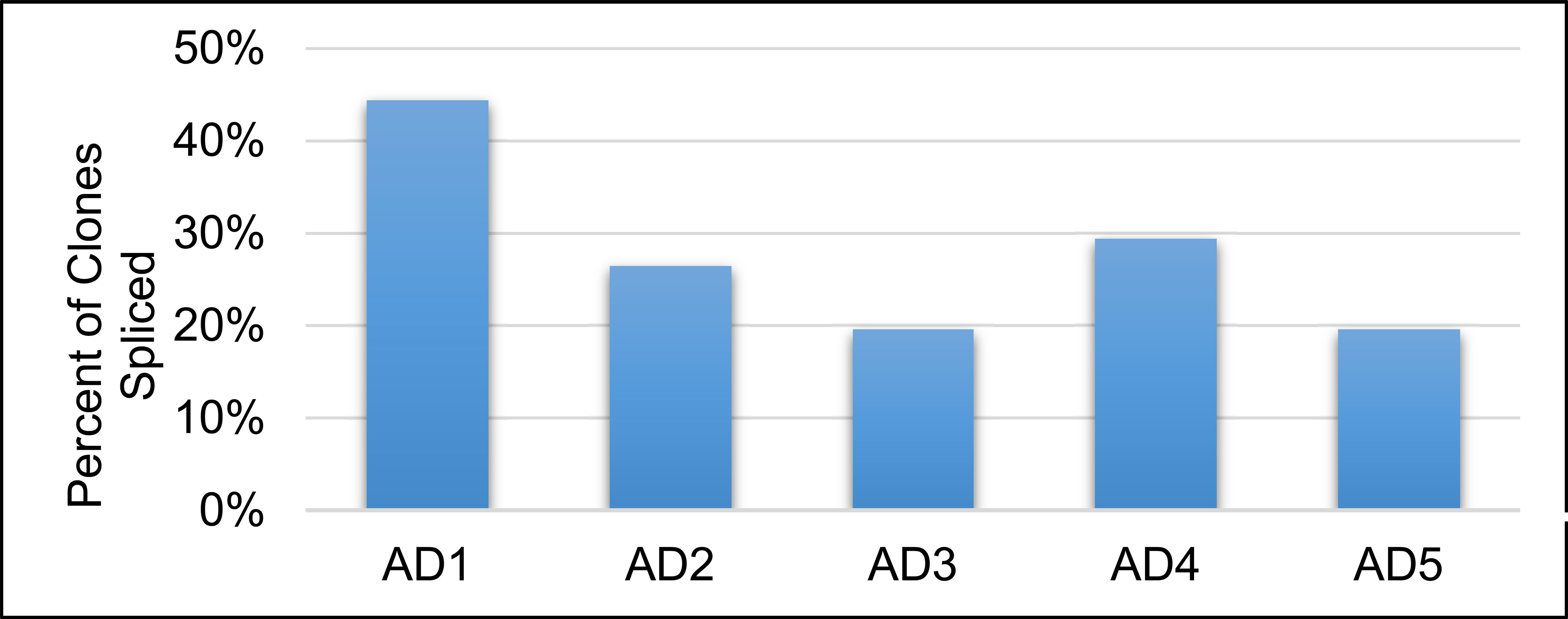

Supplement: Additional file 5: — Percentage of clones with splicing variations in each polyploid genome. The percentage of the total number of clones that contained splicing variants in each polyploid genome. Percent of splicing variants found in clones from each species. Total number of clones from each species was the sum total of the clones from bud and leaf tissue, if both were available. AD1-AD5 denote G. hirsutum, G. barbadense, G. tomentosum, G. mustelinum, and G. darwinii, respectively. [file 12870_2014_383_MOESM5_ESM.jpeg]
