# Supplementary material for: CenH3 evolution in diploids and polyploids of three angiosperm genera
Source: BMC Plant Biol. 2014 Dec 30;14:383. doi: 10.1186/s12870-014-0383-3 (PMC4308911; doi:10.1186/s12870-014-0383-3)
Supplement: Additional file 6: — Primer sequences for genomic and cDNA amplification. Primer sequences. [file 12870_2014_383_MOESM6_ESM.docx]

**Additional file 6**  Primer sequences for genomic and cDNA amplification.

| Primer Name | Sequence | Location |
| --- | --- | --- |
| 33-sense | 5’-CAAGCGTATCCGTTGCAGGTTTGT-3’ | 5' UTR |
| 35-sense | 5’- AGCGTATCCGTTGCAGGTTTGTAG-3’ | 5' UTR |
| 3041-antisense | 5’-AGGCGGTGTAGGTTTCAAGAACCA-3’ | 3'UTR |
| 3047-antisense | 5’ AGCTGAAGGCGGTGTAGGTTTCAA-3’ | 3'UTR |
| 3048-antisense | 5’- TAGCTGAAGGCGGTGTAGGTTTCA-3’ | 3'UTR |
| 3051-antisense | 5’- AGTTAGCTGAAGGCGGTGTAGGTT-3 ’ | 3'UTR |
| 2250-antisense | 5’-GCACACAGCATTGCATCTCCAAAC-3’ | Internal |
| 654-sense | 5’- TGGCTGGCTAAGTAGAGTGAGGAA-3’ | Internal |
| 1607-antisense | 5’-TGCTGCATAATTCGCGCAGAGATG-3’ | Internal |
| 1299-sense | 5’-CAAATCTACTCGTTCCTGCTGCCA-3 ’ | Internal |
| 20-sense | 5’- CCGCTGCTAAGAAACCAAGA -3’ | cDNA |
| 29-sense | 5’- AGAAACCAAGACGGAAGCC-3’ | cDNA |
| 357-antisense | 5’- CACAGCATTGCATCTCCAAAC-3’ | cDNA |
| 417-antisense | 5’-TCGCCTTGCCAACTGAATA-3’ | cDNA |
