# Supplementary material for: CenH3 evolution in diploids and polyploids of three angiosperm genera
Source: BMC Plant Biol. 2014 Dec 30;14:383. doi: 10.1186/s12870-014-0383-3 (PMC4308911; doi:10.1186/s12870-014-0383-3)
Supplement: Additional file 7: — Accessions downloaded from GenBank and Phytozome. Species names and accession numbers of Brassica and Oryza CenH3 sequences. [file 12870_2014_383_MOESM7_ESM.docx]

**Additional file 7** Accessions downloaded from GenBank and Phytozome.

| Species Description | Accession | Source | Reference |
| --- | --- | --- | --- |
| *Oryza australiensis* isolate EE | GQ849341.1 | GenBank | ([Hirsch, Wu et al. 2009](#_ENREF_31)) |
| *Oryza punctata* isolate BB | GQ849335.1 | GenBank | ([Hirsch, Wu et al. 2009](#_ENREF_31)) |
| *Oryza rhizomatis* isolate CC | GQ849339.1 | GenBank | ([Hirsch, Wu et al. 2009](#_ENREF_31)) |
| *Oryza australiensis* isolate BB | GQ849332.1 | GenBank | ([Hirsch, Wu et al. 2009](#_ENREF_31)) |
| *Oryza officinalis* isolate CC | HQ123577.1 | GenBank | ([Hui, Lu et al. 2010](#_ENREF_33)) |
| *Oryza sativa* japonica cultivar-group | AY438639.1 | GenBank | ([Nagaki, Cheng et al. 2004](#_ENREF_49)) |
| *Brassica oleracea* isolate BrCENH3-3 | GU166739.1 | GenBank | ([Wang, He et al. 2011](#_ENREF_79)) |
| *Brassica nigra* isolate BrCENH3-2 | GU166738.1 | GenBank | ([Wang, He et al. 2011](#_ENREF_79)) |
| *Brassica rapa* isolate BrCENH3-1 | GU166737.1 | GenBank | ([Wang, He et al. 2011](#_ENREF_79)) |
| *Arabidopsis thaliana* | NM_100019.1 | GenBank | ([Theologis, Ecker et al. 2000](#_ENREF_72)) |
| *Brachypodium distachyon* | Bradi2g21810 | Phytozome | ([Vogel, Garvin et al. 2010](#_ENREF_78)) |
| *Zea mays* | NM_001112050.1 | GenBank | ([Schnable, Ware et al. 2009](#_ENREF_58)) |
